# Supplementary material for: A cross-circulatory platform for monitoring innate allo-responses in lung grafts
Source: PLoS One. 2023 May 30;18(5):e0285724. doi: 10.1371/journal.pone.0285724 (PMC10228766; doi:10.1371/journal.pone.0285724)
Supplement: S1 Fig — a. Sus sternal punction. b. Wire position in the inferior vena cava (white arrow) visualized by ultrasound. c. Catheterization with double lumen canula. d. View of the double lumen canula in our cross-circulation set-up. (PDF) [file pone.0285724.s001.pdf]

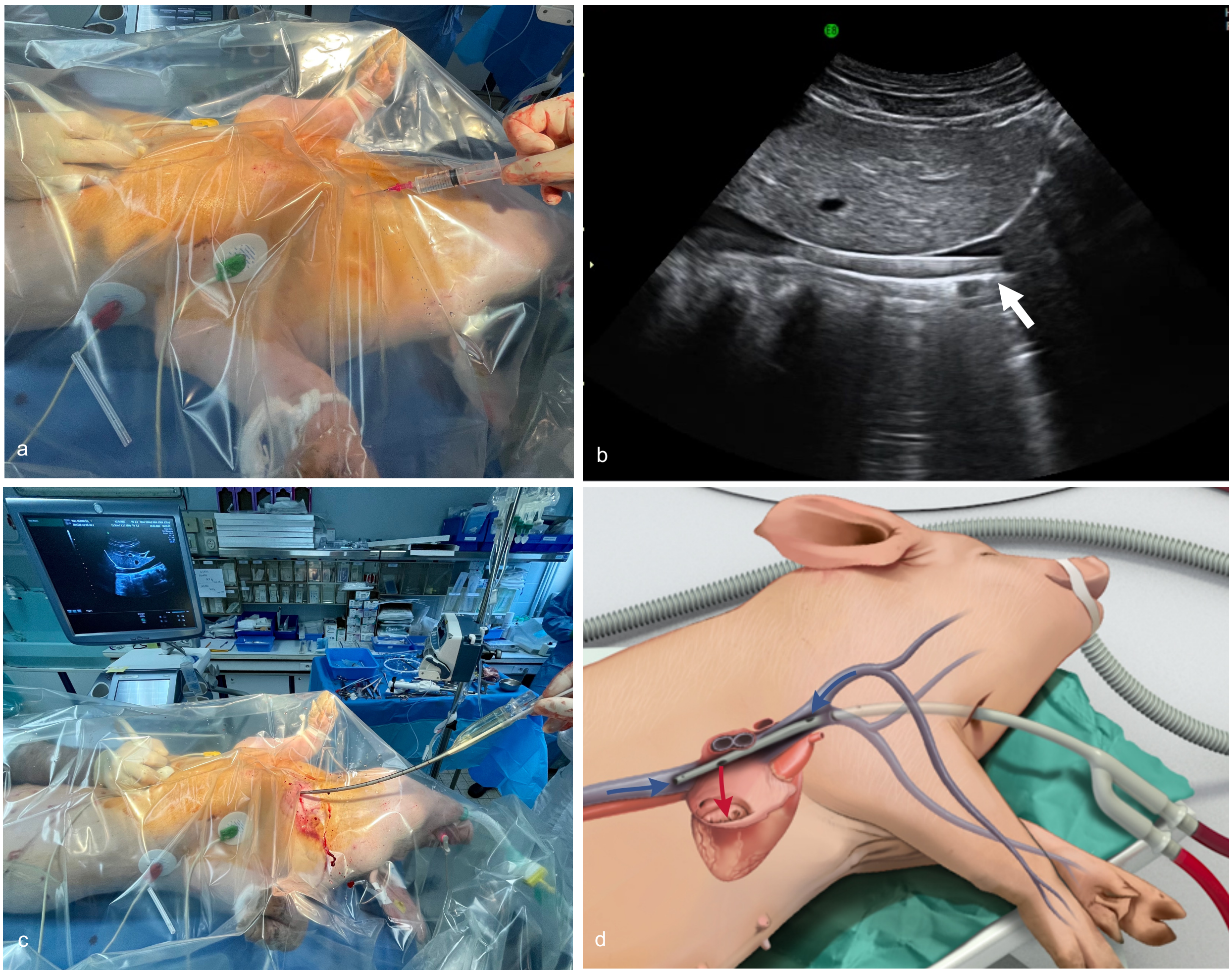

S1 Figure. Introduction of the double lumen canula 20F by the Seldinger percutaneous technique for connecting the perfusing pig circulation to the extracorporeal circuit. a. Sus sternal punction. **b.** Wire position in the inferior vena cava (white arrow) visualized by ultrasound. **c**. Catheterization with double lumen canula. **d**. View of the double lumen canula in our cross-circulation set-up.
