# Supplementary material for: A cross-circulatory platform for monitoring innate allo-responses in lung grafts
Source: PLoS One. 2023 May 30;18(5):e0285724. doi: 10.1371/journal.pone.0285724 (PMC10228766; doi:10.1371/journal.pone.0285724)
Supplement: S2 Fig — a. Lung before cross circulation initiation. b. Lung after 10 h of cross-circulation. c. General view of our cross-circulation set-up. (PDF) [file pone.0285724.s002.pdf]

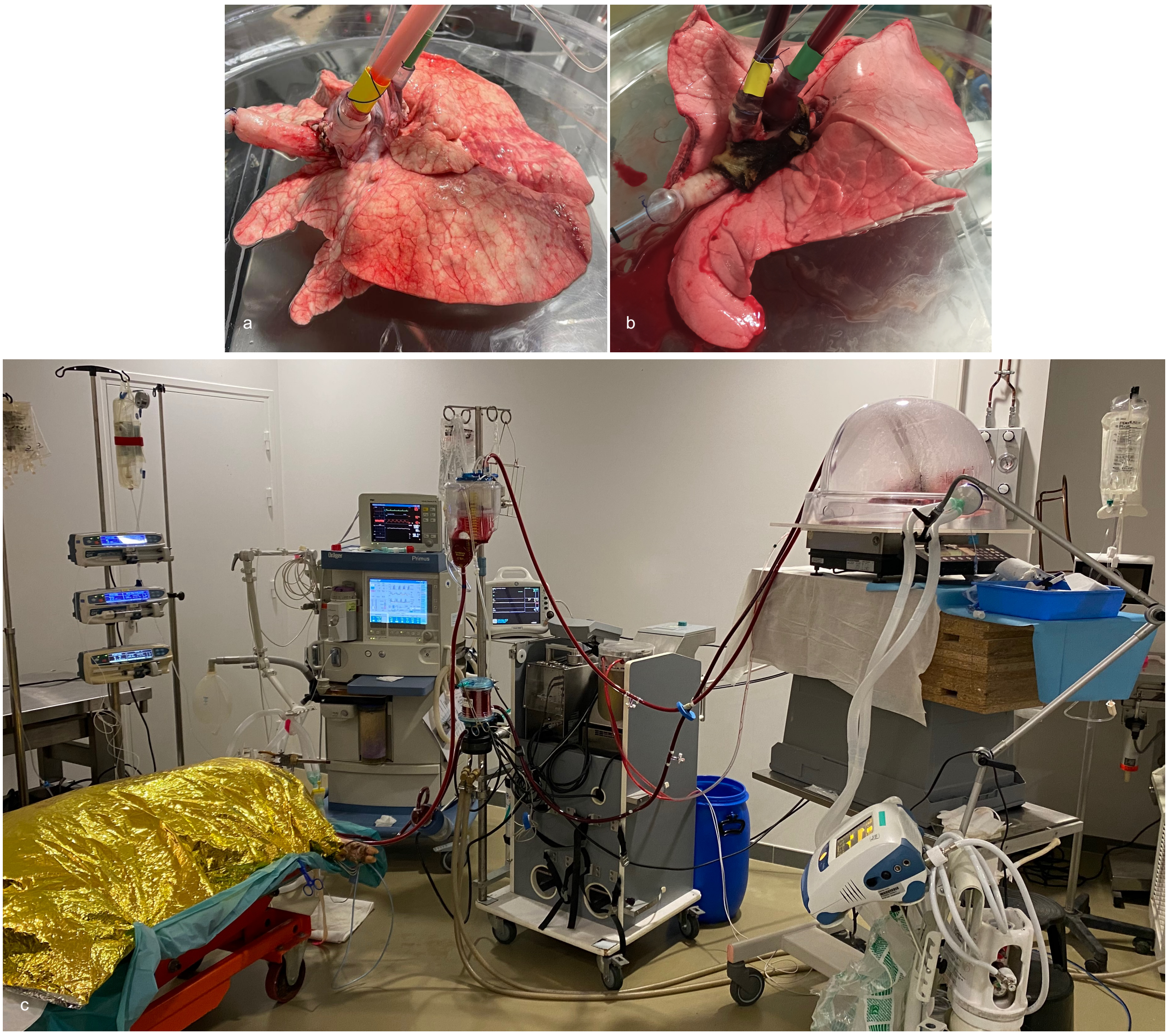

S2 Figure. Gross appearance of the lung upon cross-circulation and general view of the model. a. Lung before cross circulation initiation. **b**. Lung after 10 h of cross-circulation. **c**. General view of our cross-circulation set-up.
