# Supplementary material for: A cross-circulatory platform for monitoring innate allo-responses in lung grafts
Source: PLoS One. 2023 May 30;18(5):e0285724. doi: 10.1371/journal.pone.0285724 (PMC10228766; doi:10.1371/journal.pone.0285724)
Supplement: S3 Fig — Blood was collected 1 h before cross-circulation initiation and 1 h and 8 h post cross-circulation initiation. CFSE was injected 30 min. before cross-circulation initiation. Whole blood cells were analyzed by flow cytometry after lysis of erythrocytes. The FACS profiles of CFSE fluorescence at the different timing are superimposed onto control fluorescence obtained before cross-circulation initiation and shown as a blue histogram. The percentage of CFSEpos cells is shown. (PDF) [file pone.0285724.s003.pdf]

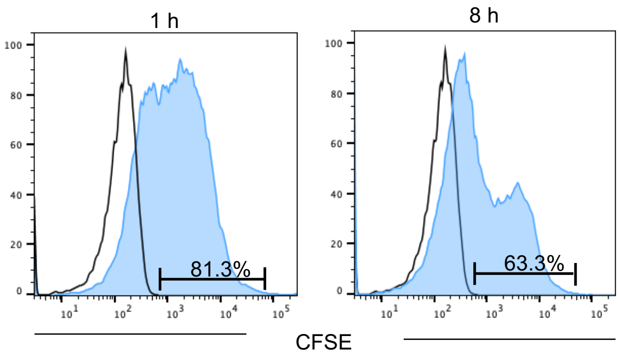

S3 Figure. Detection of CFSE staining stability in blood cells. Blood was collected 1 h before cross-circulation initiation and 1 h and 8 h post cross-circulation initiation. CFSE was injected 30 min. before cross-circulation initiation. Whole blood cells were analyzed by flow cytometry after lysis of erythrocytes. The FACS profiles of CFSE fluorescence at the different timing are superimposed onto control fluorescence obtained before cross-circulation initiation and shown as a blue histogram. The % CFSE^{pos} cells are shown.
