# Supplementary material for: A cross-circulatory platform for monitoring innate allo-responses in lung grafts
Source: PLoS One. 2023 May 30;18(5):e0285724. doi: 10.1371/journal.pone.0285724 (PMC10228766; doi:10.1371/journal.pone.0285724)
Supplement: S4 Fig — After gating on singlet and live cells, the workflow A was followed for CFSEneg cell analyses and the workflow B was followed for CFSEpos cell analyses. Except for PMNs, cells were analyzed using an intermediate gate on SSC-Alo cells to avoid noises from PMNs. PMNs, CD172Ahi (monocytic cells, MoCs), CD172Aint, CD13pos (cDC1 dendritic cell subset), NKp46pos (NK-cells), CD21pos(B-cells), CD3+ T-cells, CD3posCD4pos T-cells, CD3posCD8pos T-cell subsets are shown. (PDF) [file pone.0285724.s004.pdf]

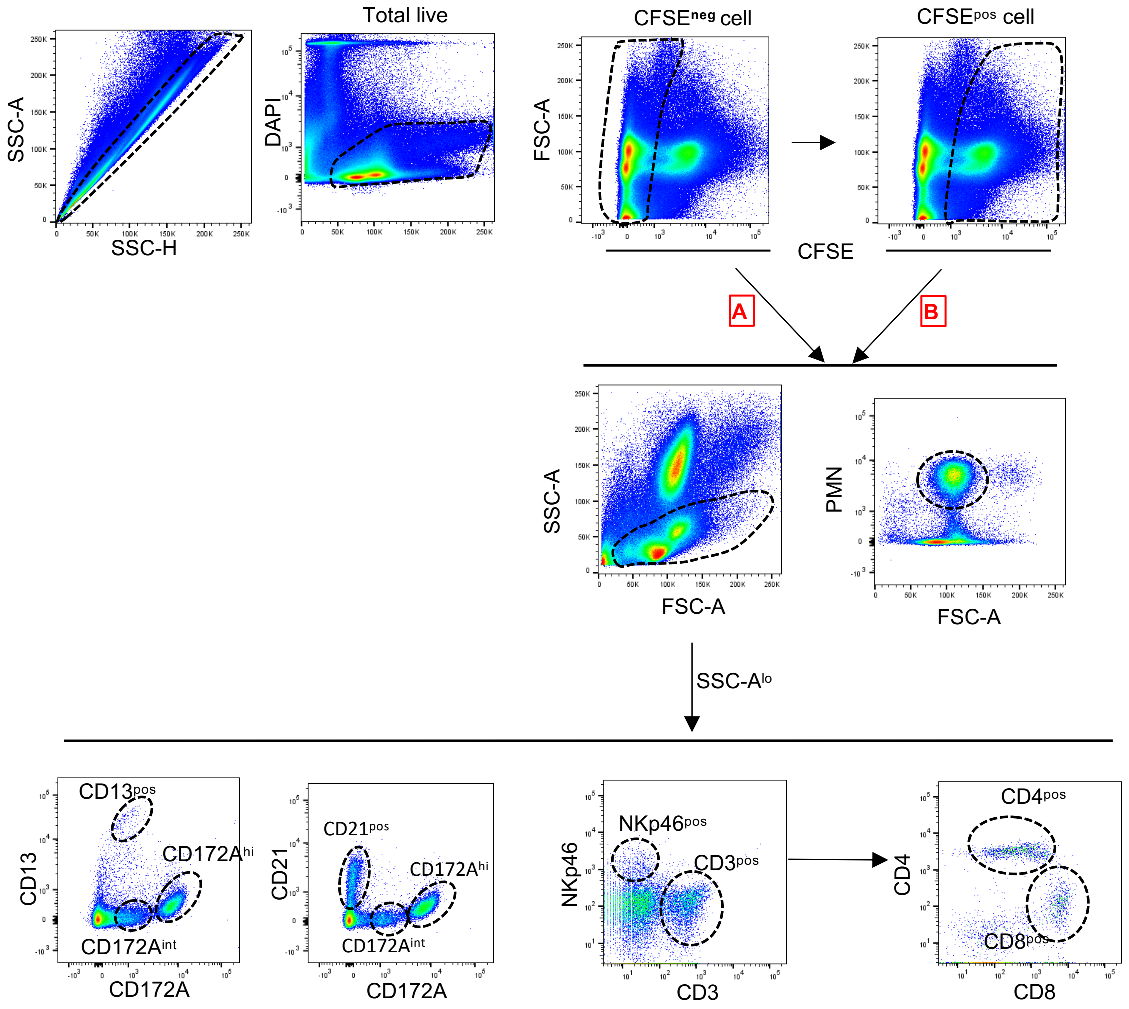

S4 Figure. Gating strategy for the pig lung immune cell analyzes. After gating on singlet and live cells, the workflow A was followed for CFSE^{neg} cells analyses and the workflow B was followed for CFSE^{pos} cell analyses. Except for PMNs, cells were analyzed using an intermediate gate on SSC-A^{lo} cells to avoid noises from PMNs. PMNs, CD172A^{hi} (monocytic cells, MoCs), CD172A^{int}, CD13^{pos} (cDC1 dendritic cell subset), NKp46^{pos} (NK-cells), CD21^{pos}(B-cells), CD3^{+} T-cells, CD3^{pos}CD4^{pos} T-cells, CD3^{pos}CD8^{pos} T-cell subsets are shown.
