# Supplementary material for: A cross-circulatory platform for monitoring innate allo-responses in lung grafts
Source: PLoS One. 2023 May 30;18(5):e0285724. doi: 10.1371/journal.pone.0285724 (PMC10228766; doi:10.1371/journal.pone.0285724)
Supplement: S5 Fig — Pig lung cells from cross-circulation experiments were analyzed for MHC class II and CD80/CD86 expression on monocytic cells at the indicated timing (0, 6, 10 h) on a representative pig shown in Fig 5 (animal represented as a "filled circle"). CFSEpos and CFSEneg monocytic cells (live SSC-AloCD172Ahi cells) were selected as shown in Supplement 6. An IgG2a isotype control (ISC) was done on a pool of lung cells from the 0, 6 and 10 h biopsies (see material and methods). The percentage of positive cells among monocytic cells is depicted. (PDF) [file pone.0285724.s005.pdf]

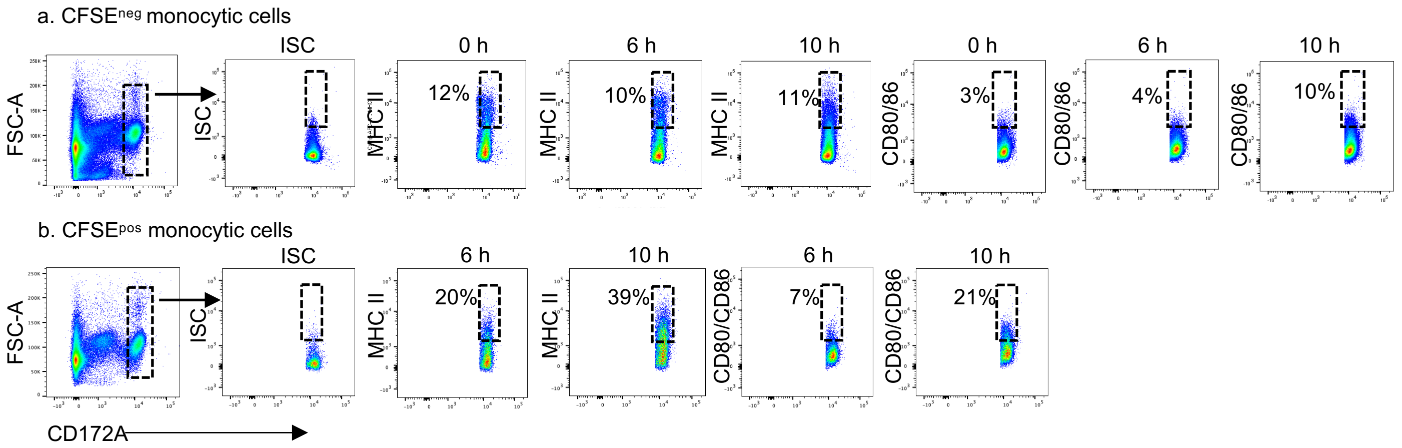

S5 Figure. MHC class II and CD80/86 expression analysis on CFSE^{neg} (a) and CFSE^{pos} (b) monocytic cells during cross-circulation. Pig lung cells from cross-circulation experiments were analyzed for MHC class II and CD80/CD86 expression on monocytic cells at the indicated timing (0, 6, 10 h) on a representative pig shown in Fig. 5 (animal represented as a "filled circle"). CFSE^{pos} and CFSE^{neg} monocytic cells **(**live SSC-A^{lo}CD172A^{hi} cells) were selected as shown in Supplement 6 . An IgG2a isotype control (ISC) was done on a pool of lung cells from the 0, 6 and 10 h biopsies (see material and methods). The percent positive cells among monocytic cells is depicted.
