# Supplementary material for: A cross-circulatory platform for monitoring innate allo-responses in lung grafts
Source: PLoS One. 2023 May 30;18(5):e0285724. doi: 10.1371/journal.pone.0285724 (PMC10228766; doi:10.1371/journal.pone.0285724)
Supplement: S6 Fig — a. Upon exclusion of granulocytes on total live lung cells, AMs were identified as CD172AhiCD163hi cells that backgated on CD172AhiSSC-Ahi cells. Upon sorting by flow cytometry, they were stained with MGG. b. Expression of MHC class II and CD80/86 was analyzed for their geometric mean intensity. The control staining with a IgG2a ISC is shown. (PDF) [file pone.0285724.s006.pdf]

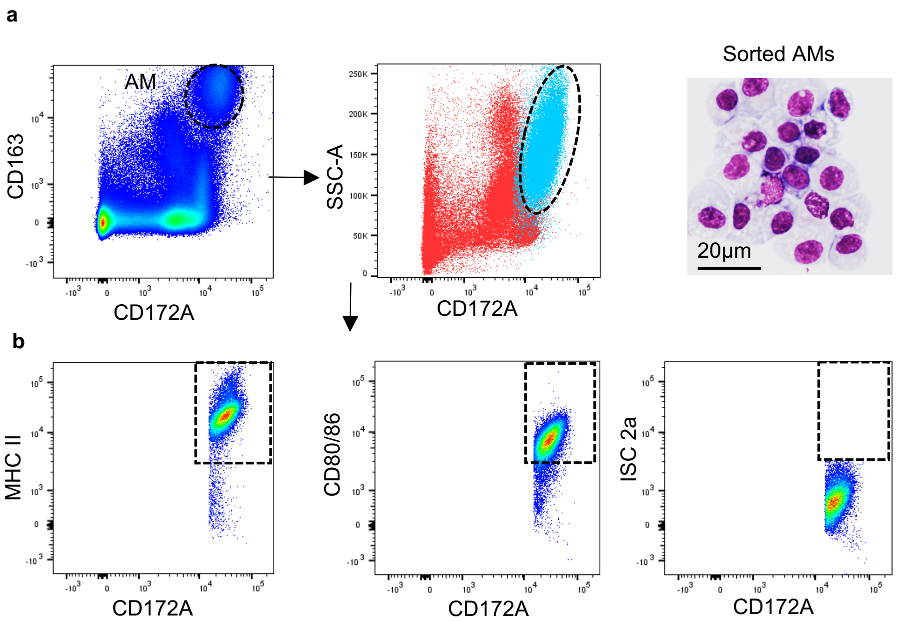

S6 Figure. Expression of MHC class II on alveolar macrophages (AMs). **a.** Upon exclusion of granulocytes on total live lung cells, AMs were identified as CD172A^{hi}CD163^{hi} cells that backgated on CD172A^{hi}SSC-A^{hi} cells. Upon sorting by flow cytometry, they were stained with MGG. **b.** Expression of MHC class II and CD80/86 was analyzed for their geometric mean intensity. The control staining with a IgG2a ISC is shown.
