# Supplementary material for: A cross-circulatory platform for monitoring innate allo-responses in lung grafts
Source: PLoS One. 2023 May 30;18(5):e0285724. doi: 10.1371/journal.pone.0285724 (PMC10228766; doi:10.1371/journal.pone.0285724)
Supplement: S7 Fig — In order to check for a possible activation effect of the non-biological surfaces of the circuit, PBMCs from a "mock" cross-circulation experiment without lung were analyzed for MHC class II and CD80/CD86 expression on monocytic cells at the indicated timing (0, 6, 10 h). CFSEpos and CFSEneg monocytic cells (live SSC-AloCD172Ahi cells) were selected as was done for the lung cells in S4 Fig. An IgG2a isotype control (ISC) was done on a pool of cells from the 0, 6 and 10 h timing. The percentage of positive cells among monocytic cells is depicted. (PDF) [file pone.0285724.s007.pdf]

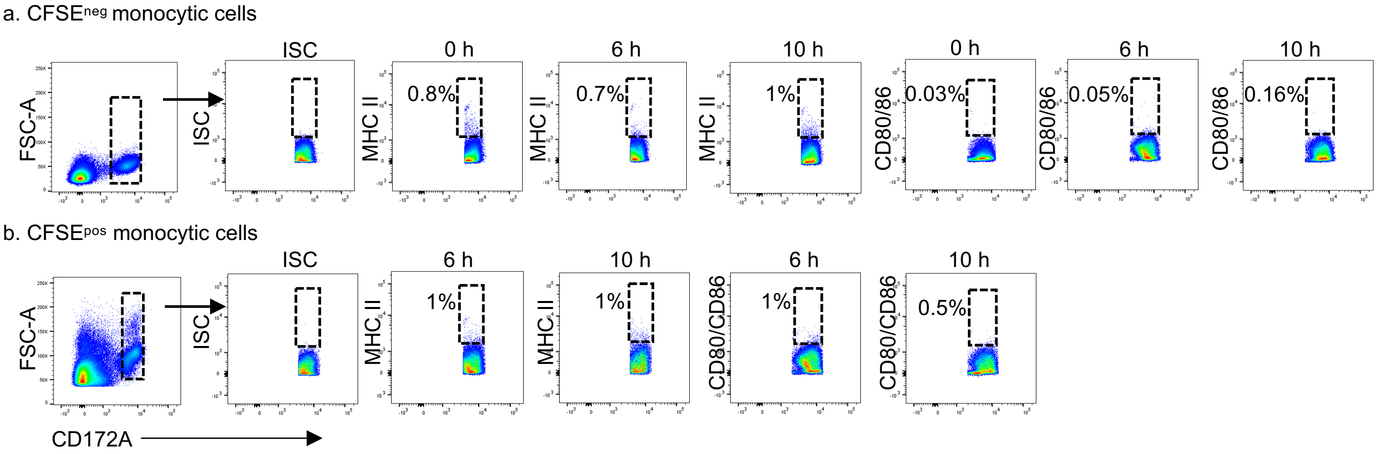

S7 Figure. MHC class II and CD80/86 expression analysis on CFSE^{neg} (a) and CFSE^{pos} (b) monocytic cells in a control experiment without donor lung. In order to check for a possible activation effect of the non-biological surfaces of the circuit, PBMCs from a "mock" cross-circulation experiment without lung were analyzed for MHC class II and CD80/CD86 expression on monocytic cells at the indicated timing (0, 6, 10 h). CFSE^{pos} and CFSE^{neg} monocytic cells (live SSC-A^{lo}CD172A^{hi} cells) were selected as done for the lung cells in S4 Figure. An IgG2a isotype control (ISC) was done on a pool of cells from the 0, 6 and 10 h timing. The percent positive cells among monocytic cells is depicted.
