# Supplementary material for: A cross-circulatory platform for monitoring innate allo-responses in lung grafts
Source: PLoS One. 2023 May 30;18(5):e0285724. doi: 10.1371/journal.pone.0285724 (PMC10228766; doi:10.1371/journal.pone.0285724)
Supplement: S1 Table — (DOCX) [file pone.0285724.s008.docx]

S1 Table. Abs (primary mAbs and secondary Abs) used in the study

| Primary antibodies | Provider | Catalog  number | Identity^1^ | Isotype (murine) | Concentration of use (mg/ml or dilution) |
| --- | --- | --- | --- | --- | --- |
| Anti-sw^2^CD13 | INRA | in-house production | T35 | IgG1 | 1/100 |
| Anti-swMHCII | WSU | PG2006 | MSA3 | IgG2a | 2 µg/ml |
| Anti-swCD172A | WSU | PG2031 | 74.22.15A | IgG2b | 2 µg/ml |
| Anti-huCD80/86 | WSU | ANC-501-020 | CTLA4-muIg^1^ | IgG2a | 5 µg/ml |
| Anti-swCD8α | WSU | PG2018 | PT81B | IgG2b | 2 µg/ml |
| Anti-swCD335 | BIO-RAD | MCA5972GA | VIVKM1 | IgG1 | 2 µg/ml |
| Anti-swCD4 | WSU | PG2013 | PT90A | IgG2a | 2 µg/ml |
| Anti-swgranulocyte | WSU | PG2045 | PG68A | IgG1 | 2 µg/ml |
| Anti-huCD21 | BD-Biosciences | 555421 | B-Ly4 | IgG1 | 5 µg/ml |
| Anti-FITC | Southern-Biotech | 6400-01 | Sheep polyclonal IgG | | 8 µg/ml |
| Conjugated primary  antibodies | Provider | Catalog number | Identity^1^ | Isotype  (murine) | Concentration of use (mg/ml or dil) |
| Anti-swCD163-PE | BIO-RAD | mca2311pe | 2A10/11 | IgG1 | 1/20 |
| Anti-swCD3-PE | BIO-RAD | MCA5951PE | PPT3 | IgG1 | 1/10 |
| Anti-swMHCII-A647 | BIO-RAD | MCA2314A647 | 2^E^9/13 | IgG2b | 5 µg/ml |
| Anti-swCD172A-PE | BD Bioscience | 561499 | 74.22.15A | IgG2b | 5 µg/ml |
| Anti-huCD80/86-PE | Ancell | 501-050 | CTLA4-muIg^1^ | IgG2a | 5 µg/ml |
| ISC-G1-PE | Invitrogen | [12-4714-82](https://www.thermofisher.com/antibody/product/Mouse-IgG1-kappa-clone-P3-6-2-8-1-Isotype-Control/12-4714-82) | P3.6.2.8.1 | IgG1 | 1/10 |
| ISC-G2a-PE | BD Bioscience | 553930 | R35-95 | IgG2a | 5 µg/ml |
| ISC-G2b-A647 | Biolegend | 400626 | RTK4530 | IgG22 | 5 µg/ml |
| Secondary Antibodies | Provider | Catalog number | Identity^1^ |  | Concentration of use (mg/ml or dil) |
| Goat anti-mu IgG2b-APC-Cy7 | Abcam | ab130791 | Goat polyclonal IgG | | 1/100 |
| Rat anti-mu  IgG1-PerCP-eFluor710-Cy5.5 | Fisher | 15361310 | Rat mAb M1-14D12 | | 1/200 |
| Goat anti-mu  IgG2a-A647 | Invitrogen | A-21241 | Goat polyclonal IgG | | 1/200 |
| Donkey anti-sh IgG-A594 | Jackson Immunoresearch | 713-586-147 | Donkey polyclonal IgG | | 5 µg/ml |

^1^ Identity corresponds either to the original clone (mAb), a fusion protein (CTLA4-muIg, i.e. human CTLA4 fused to murine IgG2a sequences), or a polyclonal IgG (species of origin). ^2^The species of the targeted molecule is either swine (sw), murine (mu), sheep (sh) or human (hu).
