# Supplementary material for: A cross-circulatory platform for monitoring innate allo-responses in lung grafts
Source: PLoS One. 2023 May 30;18(5):e0285724. doi: 10.1371/journal.pone.0285724 (PMC10228766; doi:10.1371/journal.pone.0285724)
Supplement: S2 Table — The monitored parameters are reported, 5 pigs/group for vital parameters and 4 pigs/group for blood cell counts. Values represent means ± standard deviations. A Wilcoxon matched pairs signed rank test was done between initial stabilized values for the heart, respiratory, temperature and lactate parameters (at 1 h or 2 h) and 10 h and between 0 h and 10 h for all other parameters, and showed no statistically-significant difference. BP, blood pressure. (DOCX) [file pone.0285724.s009.docx]

| **Parameter** | **Time (h)** | | | | | | | | | | |
| --- | --- | --- | --- | --- | --- | --- | --- | --- | --- | --- | --- |
|  | **0** | **1** | **2** | **3** | **4** | **5** | **6** | **7** | **8** | **9** | **10** |
| **Heart rate**  **(bpm)** | 95±24 | 92±24 | 88±13 | 81±16 | 90±22 | 90±20 | 85±19 | 92±19 | 91±5 | 95±11 | 100±14 |
| **Systolic BP**  **(mm Hg)** | 89±9 | 97±9 | 94±10 | 89±11 | 88±9 | 93±3 | 81±12 | 96±4 | 100±6 | 97±8 | 92±3 |
| **Temperature**  **(°C)** | 37.6±1.1 | 37.9±0.8 | 38.3±0.6 | 38.5±0.6 | 38.8±0.6 | 38.5±0.2 | 38.5±0.3 | 38.45±0.3 | 38.5±0.3 | 38.5±0.3 | 38.5±0.4 |
| **PH** | 7.3±0.04 |  | 7.4±0.03 |  | 7.5±0.04 |  | 7.5±0.04 |  | 7.4±0.03 |  | 7.4±0.3 |
| **Lactate  (mmol/l)** | 4.9±3.3 |  | 1.5±0.8 |  | 1.1±0.7 |  | 1.0±0.4 |  | 0.9±0.5 |  | 0.8±0.2 |
| **Creatinine (mmol/l)** | 119±25 |  | 120±1 |  | 127±14 |  | 137±17 |  | 148±24 |  | 150±26 |
| **Haemoglobin (g/dl)** | 8.9±1.6 |  | 6.7±0.4 |  | 6.9±0.2 |  | 7.3±1.5 |  | 8.2±1.5 |  | 6.8±0.6 |
| **Glucose**  **(mmol/l)** | 7.1±2.2 |  | 5.8±0.3 |  | 6.2±1.2 |  | 6.7±1.3 |  | 6.6±1.7 |  | 5.9±1.6 |
| **Activated clotting** **time  (s)** | 168±32 |  | 171±16 |  | 177±11 |  | 166±19 |  | 156±25 |  | 174±15 |
| **White Blood Cells  (10^3^/mm^3^)** | 18.2±2.8 | 12.9±2.4 |  | 14.3±2.3 |  |  | 14.3±2.3 |  |  |  | 16.8±5.8 |
| **Lymphocytes (103/mm^3^)** | 9.6±1.4 | 7.3±1.9 |  | 6.5±1.2 |  |  | 4.8±0.6 |  |  |  | 5.3±0.5 |
| **Neutrophils (10^3^/mm^3^)** | 7.6±2.9 | 4.3±0.8 |  | 7±2.8 |  |  | 9.9±6 |  |  |  | 10.1±5 |
| **Eosinophils (10^3^/mm^3^)** | 0.19±0.1 | 0.11±0.04 |  | 0.11±0.04 |  |  | 0.07±0.04 |  |  |  | 0.10±0.05 |
| **Basophils (10^3^/mm^3^)** | 0.09±0.09 | 0.04±0.02 |  | 0.08±0.04 |  |  | 0.11±0.05 |  |  |  | 0.05±0.07 |
| **Monocytes (10^3^/mm^3^)** | 0.76±0.09 | 0.54±0.12 |  | 0.75±0.12 |  |  | 0.6±0.07 |  |  |  | 1.2±0.6 |
| **Red Blood Cells (10^6^/mm^3^)** | 6.6±0.6 | 4.5±0.6 |  | 4.4±0.4 |  |  | 4.3±0.6 |  |  |  | 4.2±0.6 |
| **Platelets (10^3^/mm^3^)** | 323±49 | 237±90 |  | 209±70 |  |  | 204±88 |  |  |  | 190±60 |
| **Hematocrit**  **(%)** | 35.3±5.5 | 23.8±4.4 |  | 22.8±2.4 |  |  | 22.1±2.7 |  |  |  | 22±3.1 |

**S2 Table: Vital and biological parameters in perfusing pigs throughout 10 h of cross-circulation support.** The monitored parameters are reported, 5 pigs/group for vital parameters and 4 pigs/group for blood cell counts. Values represent means ± standard deviations. A Wilcoxon matched pairs signed rank test was done between initial stabilized values for the heart, respiratory, temperature and lactate parameters (at 1 h or 2 h) and 10 h and between 0 h and 10 h for all other parameters, and showed no statistically-significant difference. BP, blood pressure.
