# Supplementary material for: A cross-circulatory platform for monitoring innate allo-responses in lung grafts
Source: PLoS One. 2023 May 30;18(5):e0285724. doi: 10.1371/journal.pone.0285724 (PMC10228766; doi:10.1371/journal.pone.0285724)
Supplement: Attachment — Submitted filename: Response to reviewers-PlosOne.docx [file pone.0285724.s010.docx]

Jouy en Josas, March 18^th^ 2023

Dear Editor of PLOS ONE,

We have provided responses to the reviewers’ queries which you will find below. The reviewer’s text is in italic, our response is in standard font, the modified text in the manuscript is in red. We hope that our responses will be satisfactory to you and the reviewers, so that the paper can be published in PLOS ONE.

Isabelle Schwartz-Cornil, on the behalf of the authors of the manuscript PONE-D-23-01584

***Response to Reviewer 1***

*This is a very innovative study with great potential to uncover mechanisms of ischemia reperfusion injury and reveal putative targets for intervention. This area of investigation is of great interest to the lung transplant community and has translatable potential to transplantation in general. The manuscript could be enhanced through the following ways*

We thank Reviewer 1 for his/her positive general comments about our paper and his/her appreciation of the impact that this work could have in the lung transplantation field.

1*. The paper would benefit significantly from a native english speaking editor. There are countless awkward statements and grammatical errors throughout the text which can easily be corrected by a native english speaker.*

The manuscript has been corrected by a native English speaker, Mrs Polly Gobin. The changes are highlighted in red.

*2. The major deficit I see with this manuscript are the lack of different conditions to compare. There is novelty in the technique, but there is a lack of scientific rigor and the lack of varying conditions diminishes confidence that the observed immune phenotypes have a biological underpinning. By showing, for instance, variable injury phenotypes in response to prolonged ischemic times, this would vastly increase the significance of this research*.

In this paper, we describe the establishment of a model for monitoring the innate allo-responses upon lung transplantation and we show its potential to perform analysis of the recruitment of different cell types and their activation in a lung graft. As indicated by reviewer 1, this model allows to study the effects of intra-operative treatments and the effects of transplantation parameters (donation after brain death, donation after circulatory death, ventilation injuries, hemodynamic perturbations). Performing such experiments with varying conditions requires to obtain specific financial supports and to perform extensive lab work, and therefore is beyond the purpose of this paper. We are currently testing the effects of immunomodulatory drugs using this model, and experiments are ongoing. In order to take the reviewer 1’s comment into account, we added in the discussion L511 “In addition the impact of lung procurement modalities on the cellular responses could be investigated, such as donation by circulatory death versus by brain death, as well as prolonged warm ischemia”.

*3. Related to point #2 above, why did the authors chose as the sole condition: a DCD donor model with additional cold ischemia time? Arguably, a cleaner model would use a heart beating donor.*

We chose to establish the cross-circulation platform using a DCD model. DCD (Maastricht 3) is increasingly used by transplantation centers in an effort to augment the pool of donor lungs, as this type of donation provided similar clinical results as donation upon brain death according to the Toronto group [Machuca, 2015, PMID 25772069]. In addition a DCD model in the pig is more acceptable than a heart beating model by our French ethics committee.

*4. The focus of this paper is innate immune responses to ischemia reperfusion injury. I am confused as to why the authors go great lengths to introduce and explain that they specifically chose outbred pigs of differing genders to introduce an "allogeneic" component to the model. If the goal is to understand innate immunity, the model should use syngeneic transplants.*

We aimed at developing a model to study allogeneic innate responses. Therefore in the introduction, we provide references about the mechanisms that drive allogeneic innate immune responses upon transplantation, i.e. innate allo-recognition (implicating CD47/CD172A and PIR/MHC class I) and ischemia-reperfusion. However we realize that the title of the submitted version only mentioned “innate responses”, that can be confusing. Therefore we modified the title to “A cross-circulatory platform for monitoring innate allo-responses in lung grafts”. Furthermore, we add in the discussion a new comment L485 “Interestingly inbred mini-pig lines could be used to compare the innate cellular responses that are induced by ischemia-reperfusion only (syngeneic model), and by allogenicity (allogeneic model). Furthermore the cellular effects of discordant MHC class I and/or MHC class II molecules and/or AO types between the donor and perfusing pigs could be evaluated with this cross-circulatory platform”.

*5. Throughout the manuscript, the word "recruited" is used liberally to describe perfusing pig immune cells found in the allograft. This word should be used carefully as "recruited" implies an active process. Passively perfusing the lung with CSFE labeled blood is not an active biologic process...therefore CSFE+ cells in the intravascular space I would not consider as recruitment. Conversely, CSFE+ cells in the alveolar space or interstitium could be considered as "recruited" since there was a process of extravasation. Related to this comment, Fig 3 could be modified to demonstrate this with the IHC showing improved histologic resolution to identify which compartment the CSFE+ cells are in. Currently, that figure is blurry and zoomed out too far so you cannot tell histologic architecture.*

We agree that recruitment corresponds to an active process, encompassing different steps (migration due to chemoattraction, adherence to blood vessel, extravasation in the tissue). In a recent editorial in Frontiers Cell Dev Biol, Sun et al [Sun, 2021, PMID 34026766] write: “The recruitment of leukocytes includes many processes, such as the rolling and adhesion of circulating leukocytes to endothelial cells lining the blood vessel's inner wall, the transmigration from vessel’s inner wall to the tissue surrounding vessels, and migration in the tissues”. We chose to use the term “recruitment” that, we think, best corresponds to the analysis of the CFSE^+^ cells by FACS, without precluding whether the cells are located in the blood vessel or whether they underwent diapedesis. It should be noted that the lung is carefully flushed with saline at 10 h to remove most blood cells in order to mainly analyze cells that have either adhere to the blood vessels and/or entered into the tissue (thus corresponding to “recruitment”). We commented on that point L310 “The lungs had been flushed with saline at 0 and 10 h for removing most blood cells but not at 6 h, to avoid perturbing the perfusion The CFSE^+^ cells analyzed by this technique will be designated as recruited cells from the recipient pigs, not precluding whether the cells are located in the blood lumen and in the lung parenchyma.”

We provide evidences that diapedesis of CFSE^+^ cells occurred in our model as CFSE^+^ cells (originating from the blood compartment) were found in the broncho-alveolar lavage (Fig. 3c). In addition CFSE^+^ cells were found dispersed in the tissue as shown Fig. 3d (immune-histo-fluorescence on cryo-section). The pdf conversion of the PlosOne platform strongly affected the resolution of Fig. 3d. A TIFF image at high resolution was accessible on the pdf (please look at the top of the page provided by PlosOne). In order to improve clarity, we added arrows to point towards cells that appear located in the parenchyma or on the alveolar side. Cryosections from lung tissues preserve less well the lung architecture than formalin-fixed paraffine embedded sections (FFPE) therefore we agree that IHC on FFPE sections would generate better images. We spent lots of time in trying to detect CFSE^+^ cells using FFPE sections, but we could not succeed. Indeed as explained L323, we had to amplify the CFSE signal with anti-FITC antibodies for microscopy, therefore it is likely that the FFPE process modified the CFSE antigenicity, a problem often encountered with FFPE. Given the difficulty to appreciate the exact location of leukocytes in lung with standard microscopy in general, more sophisticated techniques have been developed in mouse models, such as intravital 2-photon using Qdot 655 to label the blood compartment and GFP-expressing leukocytes [Zheng, 2017, PMID 28615357] [Kurihara, 2021, PMID 33621212] [Li, 2022, PMID 35238643]. This technical approach is not amenable at present in our pig lung model.

*6. Fig 2 should include 0, 6, and 10 hr timepoints for lung histology to assess over time. For P/F ratio, is should be better defined where they are drawing samples from.*

Figure 2 now includes Time 0, 6 h and 10 h.

We used a delta P/F ratio to show the oxygenation capacity of the *ex vivo* lung graft (Figure 2b). We calculated the delta P/F ratio with the blood gas measured in the pulmonary artery and the pulmonary vein of the extracorporeal lung, as explained L189-195. For clarity, we changed the Δ symbol to delta on the figure and we modified the text: “Static compliance (Tidal Volume/(plate pressure – positive end expiratory pressure)), delta PCO2 (PA PCO2 – PV PCO2) and delta PO2/FIO2 ((PV PO2 – PA PO2) / FIO2). “

*7. Fig 5, how do you define MHCII positive cells? Need to show the FACS plots.*

The MHC class II positivity with FACS dot plots is presented in S5 Figure (supplementals of the submitted version)

*8. In the discussion I would tone down the discourse on the fallibility of rodent models to study IRI. The mouse is a much higher throughput model of lung IRI and an extremely well established model. Multiple groups throughout time have used both hilar clamp IRI models and even lung transplant models in mice to uncover the immune mechanisms of IRI. Pigs are much more expensive to use. Also, lung transplant techniques were developed in Dogs more so than pigs.*

We now changed the sentence L436 to better modulate our statements as recommended: “Therefore the pig stands as an important complementary animal model to the rodent models; whereas the latter offer incomparable advantages with targeted gene mutants for mechanistic studies, they also present anatomic limitations (size and specificities) as well as immunological properties leading to much easier establishment of tolerance to allografts and lower requirements for immunosuppressive drugs as compared to humans, therefore another translational model such as the pig is highly valuable [Golbus, 2022, PMID 36054957]”.

9*. A significant weakness in the described cross perfusion platform that needs to be mentioned by the authors is the extremely well known pro-inflammatory stimulus that is the cardiopulmonary bypass circuit (of which all the elements are in this model: pump, membrane oxygenator, reservoir, and LOTS of plastic tubing). Every clinician that uses mechanical circulatory support knows that the blood-plastic interface activates the complement system, coagulation system, and releases a host of pro-inflammatory mediators into circulation. This will certainly affect any study evaluating the innate immune response.*

Indeed we agree that a cardiopulmonary bypass circuit may have been responsible for the cell activation that we observed. Therefore we controlled for that by performing a control cross-circulation experiment without lung, and we provided a supplementary figure for illustration in the initially submitted version (S7 Fig). See L409 “Finally, we checked that the upregulation of the MHC class II and CD80/86 molecules on the perfusing pig SSC^lo^CD172A^hi^ MoCs was well induced by the cross-circulation process in the lung and not by a non-specific effect of the non-biological surfaces of the circuit. Indeed, in a pig blood connected to the circuit without lung, expression of MHC class II and CD80/86 was not increased, neither on CSFE^+^ and nor on CFSE^-^ MoCs, at 6 and 10 h (S7 Fig).

***Response to Reviewer 2***

*Very nice observational molecular work by the French Lung Transplant Group and Prof. Sage. In the current study they used 10 donors and 10 recipients and cross-circulation for 10 hours. The method cross-circulation itself is not novel but the molecular work in regards to cross-ciculation and recrutment of immune cells with a strong clinical translational potential to the donr lung has not been done by the Colombia team or any other group that has implemented the method as far as I know.*

*I have some additional questions.*

We thank reviewer 2 for his/her positive appreciation of our work.

*Why did the authors set the time to 10 hours of circulation? To make the connection between acute and chronic rejection wouldn’t it be better and more translational to run over 4 days or even more? An unharmed lung would probably last longer on cross-circulation than the injured lungs at Colombia*.

We agree that extending the duration of the cross-circulation would be valuable to study the innate allo-response over a longer time frame than 10 h. Yet our results show that cell recruitment and cell response already occur during the first 10 h. As illustrated in S3 Fig, the CFSE staining slowly decreases with time, therefore this method cannot be used for monitoring the recipient perfusing cells much beyond 10 h. This said, the cross-circulation platform could be used to study innate allo-responses over several days with a different monitoring method, such as the single-cell RNA-seq (scRNA-seq) technique, that could distinguish donor and recipient cells as they are sex-unmatched in our setting. However the scRNA-seq technique is very expensive and requires highly specialized bio-informatic resources. In addition cross-circulation over several days requires to work with conscious pigs, that implies a different experimental set-up and a much more complex logistics. In order to take this reviewer remark into account, we comment in the discussion L482 “As cross-circulation was shown to be amenable to several days using conscious pigs [Hozain, 2019, PMID 31761338], the innate allo-response could also be studied with scRNA-seq for a longer period than the 10 h duration used here (a time limit due to the progressive extinction of the CFSE staining, see S3 Fig).

*Why was a DCD lung donation model used? Would there be any differences using a beating heart donor lung? Brain dead pig models are extremely demanding to achieve but why not just use a beating heart donor?*

We chose to establish our cross-circulation platform using a DCD model. DCD (Maastricht 3) is increasingly used by transplantation centers in an effort to augment the pool of donor lungs, as this type of donation revealed to provide similar clinical results as donation upon brain death according to the Toronto group [Machuca, 2015, 25772069]. In addition a DCD model in the pig is more ethically acceptable than a heart beating model by our French ethics committee. We comment on the potential impact of DCD in the discussion L512: In addition the impact of lung procurement modalities on the cellular responses could be investigated, such as donation by circulatory death versus by brain death, as well as prolonged warm ischemia. In this paper, we used procurement upon circulatory death, that might have some effects on the type and intensity of the cellular responses that we obtained.

*Was the donor and the recipient matched according to blood type?*

There was no matching based on AO blood type nor MHC type. We used pairs of male donors and female recipients from different siblings. In the future, we could take the AO and MHC type into consideration. We comment in the discussion L482: “Interestingly inbred mini-pig lines could be used to compare the innate cellular responses that are induced by ischemia-reperfusion only (syngeneic model), and by allogenicity (allogeneic model). Furthermore the cellular effects of discordant MHC class I and/or MHC class II molecules and/or AO types between the donor and perfusing pigs could be evaluated with this cross-circulatory platform.”

*Was the lung really put on ice before establishment of cross-circulation? Perhaps it was cold storage? Either way I cannot find the ischemic time in the manuscript please add.*

In the Material and methods, we specify L125 “Lungs were placed in a sterile isolation bag with 500 ml Perfadex® and stored at 4 °C in the refrigerator. The mean duration of warm ischemia between euthanasia and cold storage was 82.8 ± 9.3 min. The mean duration of cold ischemia between cold storage and cross circulation initiation was 103.9 ± 15.6 min.”

*Did the recipient receive any immunosuppressant therapy? If not, why. Please elaborate.*

The recipient did not receive any immunosuppressive therapy on the purpose to generate a baseline of innate allo-responses with this control pig group. We are currently testing the effects of immunomodulatory medicaments using this model, and experiments are ongoing. In the discussion we comment on that point L497 “Therefore this cross-circulation platform could be used to test the effectiveness of induction drugs -which are administered at the surgical step- on this recruited monocyte activation.”

*The manuscript lacks many of the hemodynamic, and ventilatory parameters for the 10 hours. Would be interesting to see for example flow through the “donor lung”, oxygen, saturation, end-tidal CO2, compliance, and respiratory rate were continuously monitored. If I understood, it right this have been measured in this model. Perhaps it is in the supplementary file. I was unable to open those. If so, please move them to the main manuscript.*

We brought together the relevant physiological parameters for evaluating the lung graft function over time in Figure 2a-d (main manuscript). The ventilation and flow parameters through the graft were set by adjusting the respirator and pump according to the parameters explained in L166-172. Since the pigs had similar weights, we applied similar ventilation parameters (TV, rate, PEP). The lung oxygenation capacity was expressed by the delta PO2/FIO2, and the decarboxylation capacity was expressed by the delta PCO2. The graft's airway resistance was represented by the compliance, and the vascular resistance was represented by the pulmonary vascular resistance.
